# *WNT2* activation through proximal germline deletion predisposes to small intestinal neuroendocrine tumors and intestinal adenocarcinomas

**DOI:** 10.1093/hmg/ddab206

**Published:** 2021-07-19

**Authors:** Mervi Aavikko, Eevi Kaasinen, Noora Andersson, Nalle Pentinmikko, Päivi Sulo, Iikki Donner, Päivi Pihlajamaa, Anna Kuosmanen, Simona Bramante, Riku Katainen, Lauri J Sipilä, Samantha Martin, Johanna Arola, Olli Carpén, Ilkka Heiskanen, Jukka-Pekka Mecklin, Jussi Taipale, Ari Ristimäki, Kaisa Lehti, Erika Gucciardo, Pekka Katajisto, Camilla Schalin-Jäntti, Pia Vahteristo, Lauri A Aaltonen

**Affiliations:** Department of Medical and Clinical Genetics, Faculty of Medicine, University of Helsinki, FI-00014 Helsinki, Finland; Applied Tumor Genomics Research Program, Faculty of Medicine, University of Helsinki, FI-00014 Helsinki, Finland; Institute for Molecular Medicine Finland (FIMM), Helsinki Institute of Life Sciences (HiLIFE), University of Helsinki, FI-00014 Helsinki, Finland; Department of Medical and Clinical Genetics, Faculty of Medicine, University of Helsinki, FI-00014 Helsinki, Finland; Applied Tumor Genomics Research Program, Faculty of Medicine, University of Helsinki, FI-00014 Helsinki, Finland; Department of Pathology, Medicum, University of Helsinki, FI-00014 Helsinki, Finland; Institute of Biotechnology, Helsinki Institute of Life Sciences (HiLIFE), University of Helsinki, FI-00014 Helsinki, Finland; Department of Medical and Clinical Genetics, Faculty of Medicine, University of Helsinki, FI-00014 Helsinki, Finland; Applied Tumor Genomics Research Program, Faculty of Medicine, University of Helsinki, FI-00014 Helsinki, Finland; Department of Medical and Clinical Genetics, Faculty of Medicine, University of Helsinki, FI-00014 Helsinki, Finland; Applied Tumor Genomics Research Program, Faculty of Medicine, University of Helsinki, FI-00014 Helsinki, Finland; Applied Tumor Genomics Research Program, Faculty of Medicine, University of Helsinki, FI-00014 Helsinki, Finland; Department of Biochemistry, University of Cambridge, Cambridge CB2 1GA, UK; Department of Medical and Clinical Genetics, Faculty of Medicine, University of Helsinki, FI-00014 Helsinki, Finland; Applied Tumor Genomics Research Program, Faculty of Medicine, University of Helsinki, FI-00014 Helsinki, Finland; Department of Medical and Clinical Genetics, Faculty of Medicine, University of Helsinki, FI-00014 Helsinki, Finland; Applied Tumor Genomics Research Program, Faculty of Medicine, University of Helsinki, FI-00014 Helsinki, Finland; Department of Medical and Clinical Genetics, Faculty of Medicine, University of Helsinki, FI-00014 Helsinki, Finland; Applied Tumor Genomics Research Program, Faculty of Medicine, University of Helsinki, FI-00014 Helsinki, Finland; Department of Medical and Clinical Genetics, Faculty of Medicine, University of Helsinki, FI-00014 Helsinki, Finland; Applied Tumor Genomics Research Program, Faculty of Medicine, University of Helsinki, FI-00014 Helsinki, Finland; Department of Medical and Clinical Genetics, Faculty of Medicine, University of Helsinki, FI-00014 Helsinki, Finland; Applied Tumor Genomics Research Program, Faculty of Medicine, University of Helsinki, FI-00014 Helsinki, Finland; Department of Pathology, HUSLAB, HUS Diagnostic Center, Helsinki University Hospital and University of Helsinki, 00290 Helsinki, Finland; Department of Pathology, HUSLAB, HUS Diagnostic Center, Helsinki University Hospital and University of Helsinki, 00290 Helsinki, Finland; Research Program in Systems Oncology, University of Helsinki, FI-00014 Helsinki, Finland; Endocrine Surgery, Abdominal Center, University of Helsinki and Helsinki University Hospital, 00290 Helsinki, Finland; Department of Surgery, Central Finland Central Hospital, 40620 Jyväskylä, Finland; Faculty of Sport and Health Sciences, University of Jyväskylä, FI-40014 Jyväskylä, Finland; Applied Tumor Genomics Research Program, Faculty of Medicine, University of Helsinki, FI-00014 Helsinki, Finland; Department of Biochemistry, University of Cambridge, Cambridge CB2 1GA, UK; Applied Tumor Genomics Research Program, Faculty of Medicine, University of Helsinki, FI-00014 Helsinki, Finland; Department of Pathology, HUSLAB, HUS Diagnostic Center, Helsinki University Hospital and University of Helsinki, 00290 Helsinki, Finland; Department of Microbiology, Tumor and Cell Biology, Karolinska Institutet, 171 77 Stockholm, Sweden; Individualized Drug Therapy Research Program, Faculty of Medicine, University of Helsinki, 00014 Helsinki, Finland; Individualized Drug Therapy Research Program, Faculty of Medicine, University of Helsinki, 00014 Helsinki, Finland; Institute of Biotechnology, Helsinki Institute of Life Sciences (HiLIFE), University of Helsinki, FI-00014 Helsinki, Finland; Department of Biosciences and Nutrition, Karolinska Institutet, 141 83 Huddinge, Sweden; Faculty of Biological and Environmental Sciences, University of Helsinki, FI-00014 Helsinki, Finland; Department of Cell and Molecular Biology, Karolinska Institutet, 171 77 Stockholm, Sweden; Endocrinology, Abdominal Center, University of Helsinki and Helsinki University Hospital, 00290 Helsinki, Finland; Department of Medical and Clinical Genetics, Faculty of Medicine, University of Helsinki, FI-00014 Helsinki, Finland; Applied Tumor Genomics Research Program, Faculty of Medicine, University of Helsinki, FI-00014 Helsinki, Finland; Department of Medical and Clinical Genetics, Faculty of Medicine, University of Helsinki, FI-00014 Helsinki, Finland; Applied Tumor Genomics Research Program, Faculty of Medicine, University of Helsinki, FI-00014 Helsinki, Finland

## Abstract

Many hereditary cancer syndromes are associated with an increased risk of small and large intestinal adenocarcinomas. However, conditions bearing a high risk to both adenocarcinomas and neuroendocrine tumors are yet to be described.

We studied a family with 16 individuals in four generations affected by a wide spectrum of intestinal tumors, including hyperplastic polyps, adenomas, small intestinal neuroendocrine tumors, and colorectal and small intestinal adenocarcinomas.

To assess the genetic susceptibility and understand the novel phenotype, we utilized multiple molecular methods, including whole genome sequencing, RNA sequencing, single cell sequencing, RNA *in situ* hybridization and organoid culture.

We detected a heterozygous deletion at the cystic fibrosis locus (7q31.2) perfectly segregating with the intestinal tumor predisposition in the family. The deletion removes a topologically associating domain border between *CFTR* and *WNT2*, aberrantly activating *WNT2* in the intestinal epithelium. These consequences suggest that the deletion predisposes to small intestinal neuroendocrine tumors and small and large intestinal adenocarcinomas, and reveals the broad tumorigenic effects of aberrant WNT activation in the human intestine.

## Introduction

Intestinal cancer syndromes, such as Lynch syndrome, familial adenomatous polyposis, juvenile polyposis syndrome and Peutz-Jeghers syndrome are associated with elevated relative risk of small and large intestinal adenocarcinomas. An increasing amount of research has also described the familial occurrence of small intestinal neuroendocrine tumors (SI-NETs) (1–4). Occasionally, SI-NETs may also develop in individuals with *menin 1* (*MEN1*), ret proto-oncogene (*RET*) and neurofibromin *1* (*NF1*) germline mutations, and in more recent studies, inositol polyphosphate multikinase (*IPMK*) and mutY DNA glycosylase (*MUTYH*) have been suggested as predisposition genes (5,6). However, Mendelian conditions bearing a high risk to both intestinal adenocarcinomas and SI-NETs have not been described.

Studies on the three-dimensional genome organization have demonstrated that mammalian chromosomes partition into self-interacting domains, known as topologically associating domains (TADs) (7,8). Genomic regions in these interact with each other more frequently than with the surrounding regions, and genes within TADs have been shown to be often co-regulated (7,8). Disruption of a TAD border may result in aberrant genomic interactions, disturbed gene regulation and disease (9).

Here, we describe a family with multiple individuals segregating small and large intestinal epithelial cancers, including neuroendocrine tumors and adenocarcinomas. Several family members also had intestinal hyperplasias and adenomas. Using whole genome sequencing and linkage analysis, we identified a 121.1 kb heterozygous deletion at the cystic fibrosis locus (7q31.2), segregating with the intestinal neoplasms in the family. The deletion removes a TAD border between CF transmembrane conductance regulator (*CFTR*) and Wnt family member 2 (*WNT2*), inactivating the former and activating the latter in the intestinal epithelium of the deletion carriers. To our knowledge, this is the first report of TAD border disruption as a likely cause for cancer predisposition, emphasizing the value of whole genome sequencing in detecting disease-causing variants.

## Results

**Figure 1:**
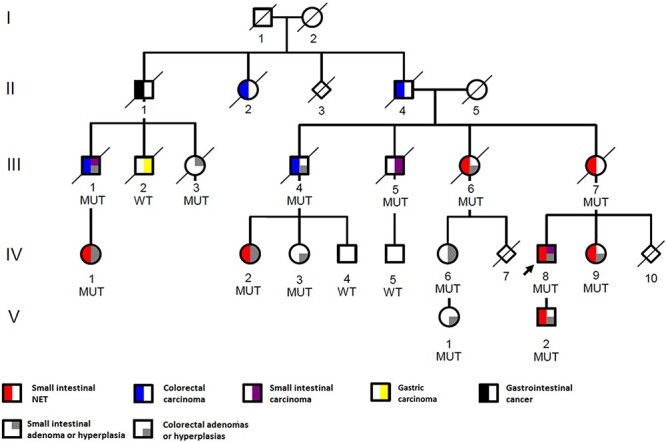
Pedigree of the family with multiple intestinal tumors. The proband (IV-8) is indicated with an arrow. Heterozygous deletion carriers are marked with MUT and wild-type allele carriers with WT sign. Squares denote males and circles females. Diamond symbol marks individuals whose gender is unknown and diagonal line marks the deceased. Generations are marked with roman numbers and individuals with Arabic numbers. The pedigree has been modified for confidentiality.

The family included 16 individuals with confirmed intestinal tumors in four generations (Fig. 1). The proband (IV-8) had a history of prolonged diarrhea, flushing and wheezing, and was diagnosed with multiple ileal neuroendocrine tumors with liver metastases and carcinoid syndrome at the age of 48. Fourteen years later, he was also diagnosed with locally metastasized duodenal adenocarcinoma. He regularly undergoes colonoscopy and numerous hyperplastic polyps and adenomas have been removed. His son (V-2), sister (IV-9), mother (III-7), aunt (III-6), cousin (IV-2) and second cousin (IV-4) have also been diagnosed with multiple (in some cases up to hundreds) SI-NETs (Fig. 1, Supplementary Material, Table S1). Five of these patients had local or distant metastases, and all examined SI-NETs were well differentiated, gradus 1 (Ki 67; < 2%) tumors and stained positive for synaptophysin and chromogranin A (Supplementary Material, Tables S1 and S2).

Other intestinal cancers in the family include small intestinal adenocarcinoma (ampulla of vater: III-1 and III-5) and colorectal carcinoma (unspecified rectal carcinoma: II-2, and adenocarcinomas: II-4, III-1 and III-4). Several individuals had been diagnosed with benign intestinal lesions, including ampulla of vater adenoma (III-3 and IV-6), ileal hyperplasia or adenoma (IV-2 and IV-1), as well as hyperplastic polyps and adenomas of the large intestine (III-1, III-4, III-6, IV-1, IV-2, IV-3, IV-6, IV-9, V-1 and V-2). These were often multiple, and in the clinical records of IV-9, they were referred to as polyposis (Supplementary Material, Table S1). One gastrointestinal cancer with unspecified location (II-1) and one gastric adenocarcinoma (III-2) was also present in the family (Fig. 1). The family members are regularly followed-up at the clinic, and certain tumor diagnoses of IV-1, IV-3, IV-6, IV-8, V-1 and V-2 have been made during the follow-up. Details of the tumor diagnoses and other clinical data are summarized in Supplementary Material, Table S1.

To understand the molecular basis of the intestinal tumor predisposition in the family, we first focused on the patients with multiple SI-NETs, as this rare phenotype carried a low possibility of phenocopies. That is, individuals with this disease were likely to carry a predisposing change, rather than being incidental. To map the genomic regions shared by the SI-NET patients, we performed linkage analysis, which resulted in 239.3 centimorgan (cM) of candidate genomic regions with positive logarithm of the odds (LOD) score (Supplementary Material, Table S3). To assess the single nucleotide variants and small insertions and deletions within these regions, we whole genome sequenced the germline DNAs of four SI-NET patients (Supplementary Material, Table S4), and scrutinized the shared variants. After removing variants with minor allele frequency (MAF) > 0.001 in the Genome Aggregation Database (gnomAD, http://gnomad.broadinstitute.org/), and in the Finnish individuals in gnomAD, a single shared protein coding variant remained (Supplementary Material, Table S5). This missense variant (p.Arg362Trp, rs756287596) in *Tumor Suppressor Protein 73* (*TP73*) was *in silico* predicted damaging (Polyphen2: probably_damaging, score 1.000; PROVEAN: deleterious, score − 6.57). However, when we screened the variant in the remaining three SI-NET patients (III-7, IV-1, and IV-2), it was not detected in IV-1, although multiple independent samples were studied. We also screened the variant in healthy geographically matched control individuals and it was present in two out of the 365 individuals (MAF = 0.003).

**Figure 2:**
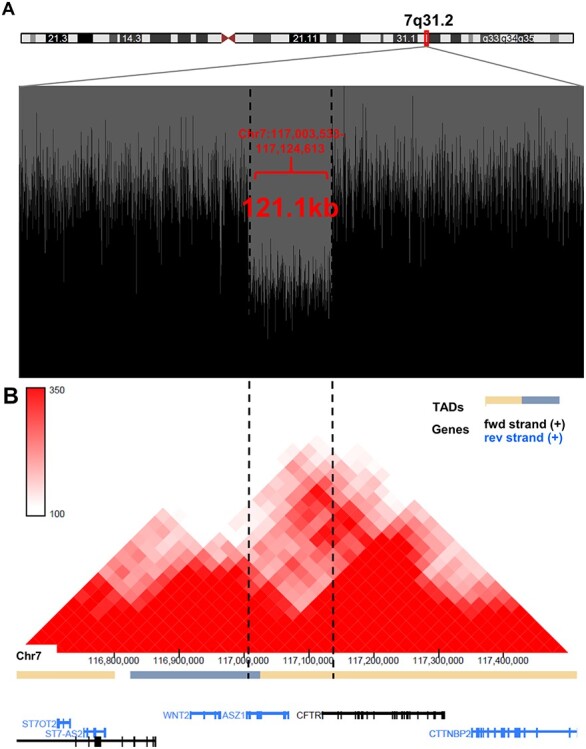
Deletion in 7q31.2 removes a topology associating domain border. (**A**) Drop in the sequencing reads (black) in the whole genome sequence data at the site of the heterozygous deletion. Whole genome sequence reads are visualized with BasePlayer (16). (**B**) The deletion spans a TAD border between *CFTR* and *WNT2*. The Hi-C interaction heatmap (red triangles) was obtained from 3D Genome browser (http://3dgenome.fsm.northwestern.edu/view.php), and is based on the data generated from GM12878 (Lieberman-raw; 25 kb resolution, hg19) (10).

We next studied the whole genomes for shared rare structural variants (SVs) and identified a heterozygous 121.1 kb deletion in 7q31.2 (Fig. 2). The deletion segregated in all seven SI-NET patients and the exact position of the deletion (Chr7:117003533-117 124 613; GRCh37) was determined by Sanger sequencing (Supplementary Material, Fig. S1). The germline whole genomes of 327 Finnish control individuals were all negative for the deletion, as were 313 additional healthy geographically matched controls screened for the deletion. The deletion was further screened in in the extended pedigree and was shown to segregate perfectly also with the other intestinal neoplasms in the family (Fig. 1). We additionally screened the deletion in 49 unrelated Finnish SI-NET patients, of which four had a first degree relative with SI-NET, and 29 had multiple tumors at the time of diagnosis, but identified none of them to harbor the deletion.

The deletion spans from the 3’UTR of Ankyrin repeat, SAM and basic leucine zipper domain containing 1 (*ASZ1*) to the first intron of *CFTR* removing exon 1 (Fig. 2). Moreover, the deletion overlaps a previously characterized TAD boundary (10) located within *ASZ1*, separating *CFTR* and *WNT2* in two separate TADs (Fig. 2B). To study the effect of the deletion on the regulation of nearby genes, we extracted and sequenced the RNAs of four small intestinal samples (three neuroendocrine tumors and a normal ileum sample) from a deletion carrier (V-2), and compared the data to small intestinal control tissue data (*n* = 4, Supplementary Material, Table S4) (11). We scrutinized the entire chromosome 7 candidate genomic region (Chr7:105323488-123 764 045; GRCh37), including *ASZ1*, *CFTR* and *WNT2* and encompassing altogether 72 protein coding genes. The most differentially expressed gene was *WNT2* (log2 fold change: 4.9, false discovery rate [FDR] adj. *P*-value 1.4E-11)*. CFTR* expression was slightly reduced (log2 fold change: −1.1, FDR adj. *P*-value: 0.060) and *ASZ1* expression was not detected (Supplementary Material, Table S6). When we compared tumors to normals (V-2 SI-NETs compared to V-2 normal sample and control data), *WNT2* expression was not significantly altered (log2 fold change: 0.63, adj. *P*-value: 0.40), whereas *CFTR* expression was slightly decreased (log2 fold change: −0.84, FDR adj. *P*-value: 0.0028; Supplementary Material, Table S7).

To further study the *WNT2* up-regulation, we extracted RNA from colonoscopy biopsies from three deletion carriers (IV-1, IV-6 and V-2) and RNA sequenced the samples. When we compared the colonoscopy biopsies (tumors and normal tissues from the deletion carriers) to colon tissue controls (*n* = 5, Supplementary Material, Table S4), *WNT2* showed again striking up-regulation and was the sixth most variable gene among all protein coding genes (log2 fold change: 9.0, FDR adj. *P*-value: 3.29E-115; Supplementary Material, Table S8).

To confirm that the observed *WNT2* up-regulation resulted due to the deletion, we studied the allele-specific expression of *WNT2*. Two deletion carriers (IV-1 and V-2) had heterozygous germline SNPs (rs2024233, rs3840660 and rs4730775) in the 3’UTR of *WNT2.* We inspected the allele balance of these SNPs in the RNA sequenced intestinal samples of IV-1 and V-2, and observed monoallelic *WNT2* expression. This was further confirmed by cDNA sequencing (Supplementary Material, Fig. S2a). The same applied to *CFTR* at the site of two informative germline SNPs rs121909046 and rs213950 (Supplementary Material, Fig. S2b). Further haplotype analysis confirmed that the monoallelic *WNT2* expression originated from the deletion harboring allele, whereas the monoallelic *CFTR* expression from the unaffected allele. Of note, we also studied intestinal biopsies of a non-deletion carrier (IV-5), who also harbored heterozygous germline SNPs in *WNT*2 and *CFTR*. As expected, we observed biallelic expression of *CFTR* in his samples (Supplementary Material, Fig. S2b). Despite various efforts, we failed to amplify *WNT2* in his samples, likely due to low basal expression of *WNT2* in the normal gastrointestinal tract (https://gtexportal.org/, v7).

**Figure 3:**
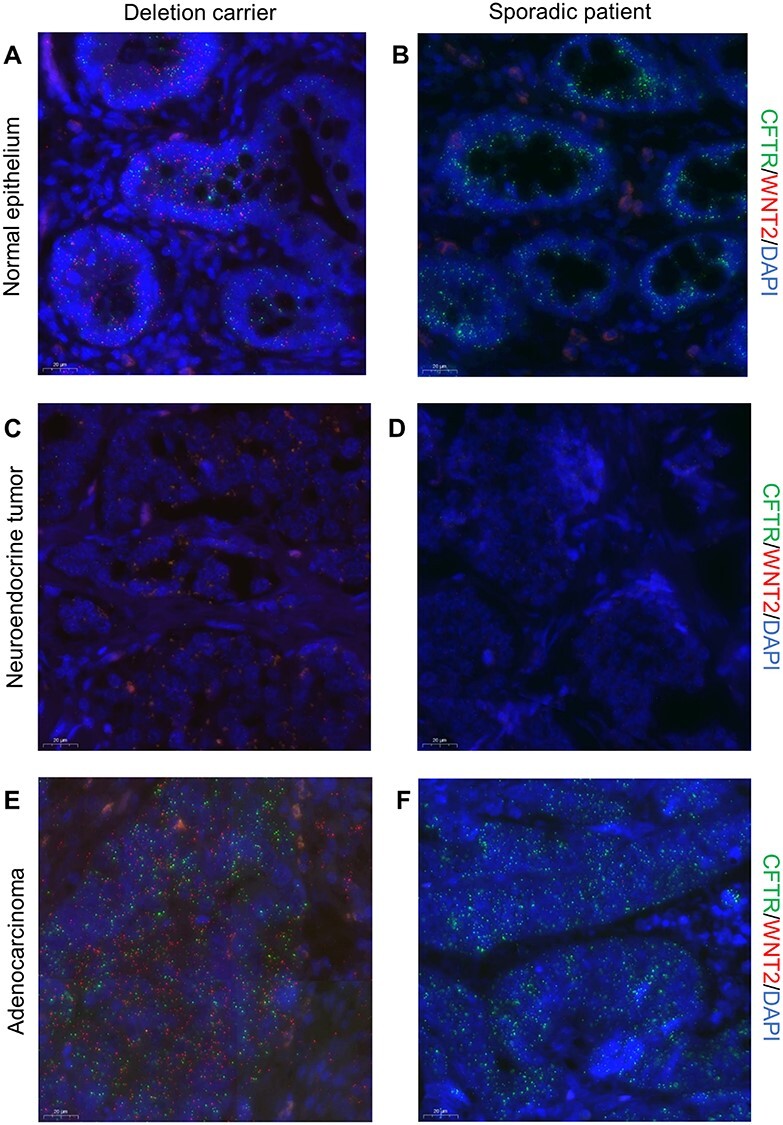
*WNT2* is expressed in the normal intestinal epithelium and adenocarcinomas of the deletion carriers. (**A-F**) RNA *in situ* hybridization of *CFTR* (green) and *WNT2* (red) in normal intestinal epithelium and tumors of a deletion carrier and a sporadic patient. The nuclei are stained with DAPI (blue). Identical image settings were used for the compared tissue types. Following signal intensity settings were used: DAPI 90, Cy3 150 and Cy5 30 (**A, B**), DAPI 90, Cy3 150, Cy5 30 (**C, D**), DAPI 80, Cy3 90 and Cy 50 (**E, F**). **A**, Normal small intestinal epithelium of a deletion carrier and **b**, a sporadic patient shows uniform expression of *CFTR*. **A,**  *WNT2* is expressed only in the intestinal epithelium of the deletion carrier. **C**, Small intestinal neuroendocrine tumor of a deletion carrier and **D,** a sporadic patient displays no expression of *CFTR* or *WNT2*. **E,** Colorectal adenocarcinoma of a deletion carrier and **F**, a sporadic patient show uniform expression of *CFTR*. **E**, *WNT2* expression is present only in the adenocarcinoma of the deletion carrier.

To study the morphological location of *WNT2* and *CFTR* expression in the tumors of the deletion carriers, we performed RNA *in situ* hybridization. Tissue samples from sporadic cases were stained as comparison. The sporadic subjects were confirmed not to have close relatives diagnosed with the same tumor type and not to carry the 7q31.2 deletion. *CFTR* expression was abundant in the intestinal epithelium of the deletion carriers and sporadic cases (Fig. 3A-B). However, *WNT2* expression was detected only in the intestinal epithelium of the deletion carriers (Fig. 3A-B). SI-NETs were devoid of *CFTR* and *WNT2* expression (Fig. 3C-D), whereas both genes were expressed in the adenocarcinomas (Fig. 3A) and colorectal adenomas (Supplementary Material, Fig. S3) of the deletion carriers.

**Figure 4:**
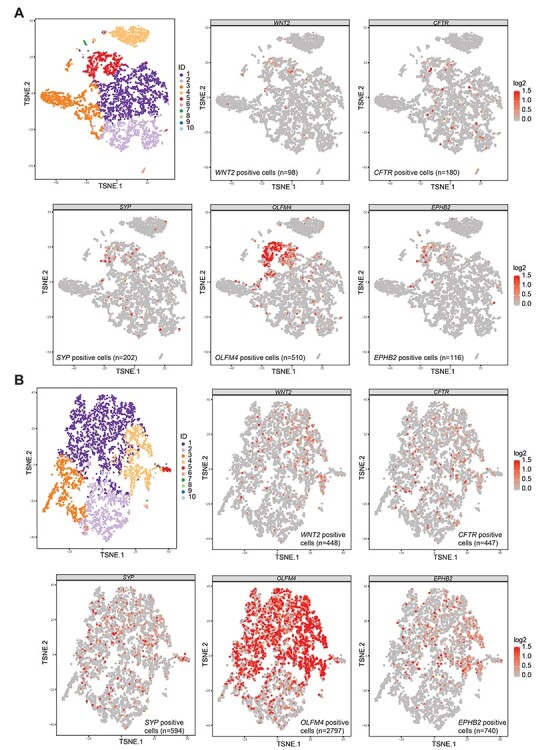
*WNT2* expression is enriched among the intestinal crypt cells and enteroendocrine cell marker *SYP*. (**A**) Upper panel left: K-means 10 clustering of the single cell gene expression data of the normal ileum sample of patient IV-1. Cluster IDs: 1–2 (epithelial cells), 3 (cells with low gene expression of mainly mitochondrial genes), 4 (immune cells, mainly T and B lymphocytes), 5 (crypt cells), 6 (complement components expressing cells), 7–8 (miscellaneous), 9 (adipose cells), 10 (muscle cells). Upper panel middle, right and lower panel: Single cells from the normal ileum expressing *WNT2*, *CFTR, SYP*, *OLFM4* and *EPHB2*. Color scale represents the normalized and log2-transformed expression of the gene. (**B**) Upper panel left: K-means 10 clustering of the single cell gene expression data of the ileal adenoma of patient IV-1. Cluster IDs: 1–2 (epithelial cells), 3 (immune cells, mainly B lymphocytes), 4 (crypt cells), 5 (enteroendocrine/secretory cells), 6 (immune cells, likely T lymphocytes), 7–10 (miscellaneous cells). Upper panel middle and right and lower panel: Single cells from the ileal adenoma expressing *WNT2*, *CFTR, OLFM4* and *EPHB2*. Color scale represents the normalized and log2-transformed expression of the gene.

To further examine if *WNT2* and *CFTR* were expressed by the same cells, we performed single cell RNA-sequencing and gene expression analysis of normal ileum and ileal adenoma of a deletion carrier (IV-1, Supplementary Material, Fig. S4). Although initially suspected as SI-NET in the computer tomography, the tumor was later in the pathological review confirmed to be an ileal adenoma with no evidence of neuroendocrine tumor. Compatible with our hypothesis that the *WNT2* up-regulation stems from the TAD breakage, causing *CFTR* regulatory elements to drive *WNT2* expression, cells expressing both *WNT2* and *CFTR* were enriched (normal sample, *P*-value: 0.0085, 95% confidence interval [CI] 0.0011–0.0042, adenoma sample, *P*-value: 8.65E-12, 95% CI 0.016–0.024, two-sided exact binomial test). However, in the normal ileum, *WNT2* expression was mostly present in the predicted crypt cells, whereas *CFTR* expression was seen in many different epithelial cell types (Fig. 4). In the adenoma, both genes were expressed across different cell types (Fig. 4). We also studied if *WNT2* expression was associated with expression of enteroendocrine cell/neuroendocrine tumor markers chromogranin A (*CHGA*) and synaptophysin (*SYP*). *CHGA* expression was detected only in 1/97 and 2/448 *WNT2* expressing cells in the normal ileum and ileal adenoma, respectively. *SYP* expression was more prevalent in the samples, and was shown to be associated with *WNT2* expression, especially in the adenoma cells (normal sample *P*-value: 0.0025, 95% CI 0.0014–0.0048; adenoma sample *P*-value: 8.864E-07, 95% CI 0.017–0.025, two-sided exact binomial test, Fig. 4). It was of particular interest to see that the expression of *OLFM4* (olfactomedin 4) and *EPHB2* (EPH Receptor B2), both established intestinal stem cell markers*,* were strongly associated with *WNT2* expressing cells (normal sample: *OLFM4 P*-value: 2.20E-16, 95% CI 0.010–0.017; *EPHB2 P*-value: 6.588E-12, 95% CI 0.0028–0.0070; adenoma sample: *OLFM4 P*-value: 6.99E-12, 95% CI 0.074–0.090; *EPHB2 P*-value: 2.2E-16, 95% CI 0.027–0.037, two-sided exact binomial test) (Fig. 4) suggesting autocrine regulation of WNT activity in the stem cells (Fig. 4).

**Figure 5:**
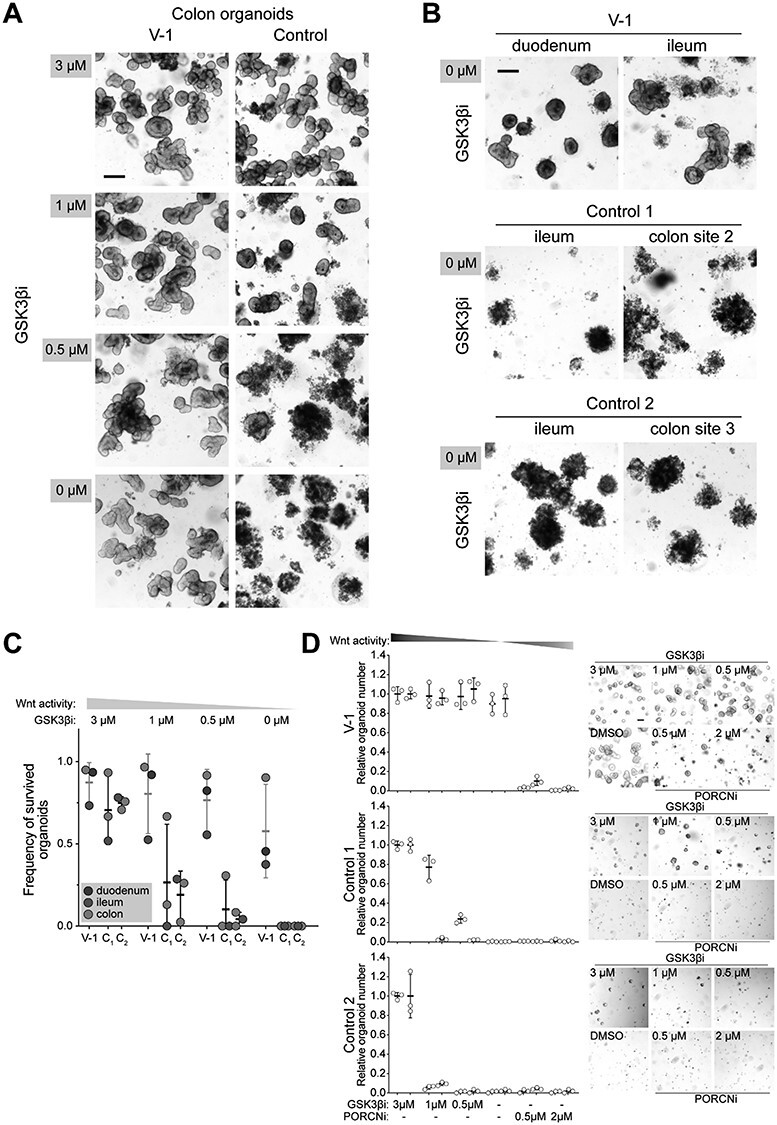
7q31.2 deletion carriers’ intestinal organoids are maintained without *in vitro* activation of the canonical WNT pathway. (**A**) Representative images of the colonic organoids grown four days in reducing concentration of GSK3β inhibitor (GSK3βi) in the culture media. Scale bar 200 μm. (**B**) Duodenal and ileal organoids from 7q31.2 deletion carrier (V-1), and ileal and colonic organoids from two controls cultured in the absence of GSK3βi. Scale bar 200 μm (**C**) Frequency of the surviving organoids after four days in reducing concentration of GSK3βi, (**D**) Relative number and representative images of single cells derived organoids grown in reducing GSK3βi and increasing porcupine inhibitor (PORCNi) concentrations. Images and quantification are done six days post plating. Quantitative data collected from three replicates of two different passages derived from the same organoid culture (V-1). For controls the data was collected from three replicates of two different colon organoid cultures. Scale bar 100 μm.

Finally, to investigate if the aberrant WNT2 secretion could drive the neoplastic growth, we cultured organoids from intestinal biopsies of a deletion carrier (V-1), and two age and sex matched controls (Supplementary Material, Table S4). Intestinal organoids require activation of the canonical WNT pathway for normal growth, which can be obtained by adding GSK3β inhibitor (CHIR99021, GSK3βi) into the culture media. To test if the deletion carriers’ organoids could survive without exogenous WNT pathway activation, organoids were grown in lowering concentrations of GSK3βi. As demonstrated in Figure 5, the deletion carriers’ organoids remained vital even in the absence of GSK3βi. The effect was present regardless of the intestinal location of the organoids, but most evident in the colonic organoids (Fig. 5B-C). Strikingly, WNT activation in deletion carriers’ cells was able to sustain clonal growth of single cells (Fig. 5D). To confirm that the observed growth benefit was due to excess production of WNT ligands, we utilized porcupine inhibitor (IWP-2, PORCNi), which prevents acylation and secretion of WNT ligands (12). PORCNi abolished the organoid growth in GSK3βi free media (Fig. 5D). Together these data demonstrate that aberrant growth in deletion carriers’ organoids was due to excessive production of WNT ligand.

Taken together, our genetic and functional data support the hypothesis that the deletion at 7q31.2 leads to aberrant activation of *WNT2* resulting in niche independent growth of intestinal stem cells.

## Discussion

High relative risk to intestinal adenocarcinoma is a characteristic feature of several well established intestinal cancer syndromes. SI-NETs have also been reported to cluster in families (1–4), and occasionally manifest in individuals with Mendelian disorders, such as Multiple endocrine neoplasms (MEN1) and neurofibromatosis 1 (NF1). To the best of our knowledge, Mendelian conditions causing a high risk to intestinal adenocarcinomas as well as neuroendocrine tumors have not been previously described.

By studying a family with 16 affected individuals, we identified a heterozygous deletion at the cystic fibrosis locus (7q31.2) perfectly segregating with the intestinal cancers in the family. The deletion was further screened in 49 additional SI-NET patients, but no additional deletion carriers were found, and it is likely that the particular deletion is private to the family. Thorough screening of the locus, with methods that can detect other TAD breaking chromosomal alterations and *WNT2* activating mutations is warranted in patients with similar phenotype to the family.

The deletion disrupts the TAD border between two genes (*CFTR* and *WNT2*) and leads to aberrant expression of *WNT2* from the deletion allele in the intestinal epithelium. The genetic findings, supported by the functional experiments, suggest a new cancer syndrome caused by aberrant activation of *WNT2* that predisposes to SI-NETs and intestinal adenocarcinomas.

*WNT2* encodes a secreted signaling protein normally expressed in the placenta and in adult tissues primarily in the lung. It can activate the canonical WNT signaling pathway, crucial for the intestinal stem cell regulation. Aberrant activation of the canonical WNT pathway is considered an essential early event in the colorectal carcinogenesis, and overexpression of *WNT2* has been reported in esophageal, colorectal and gastric cancers (13–15).

*CFTR* encodes a chloride channel that regulates ion and water secretion and absorption. Its expression is highly tissue specific, and is most abundant in the intestinal epithelium. In the esophagus and stomach, *CFTR* expression is low (https://gtexportal.org/, v7). *CFTR* expression is regulated by well-documented tissue-specific enhancers located in introns 1, 10 and 11 that interact with the *CFTR* promoter via chromosomal looping (16,17). We hypothesized that by removing the TAD border between *CFTR* and *WNT2* and the *CFTR* promoter, the deletion rewires the intestine specific enhancers of *CFTR* to interact with the *WNT2* promoter*,* aberrantly activating its secretion in the non-neoplastic intestinal epithelium. Thus, cells expressing *WNT2* would be expected to overlap with those expressing also the normal *CFTR* allele, which indeed was the case.

However, it is also expected that the set of transcription factors required for *WNT2* expression and secretion would be somewhat different from those required for *CFTR* expression. Such factors might in particular be available in the intestinal crypt cells, as suggested by the co-expression of *WNT2* with intestinal stem cell markers *OLFM4* and *EPHB2*. Interestingly, *WNT2* expression was also associated with enteroendocrine/neuroendocrine tumor marker *SYP* expressing cells especially in the intestinal adenoma of IV-1, which was pathologically confirmed to be tubulovillous adenoma with high-grade dysplasia and not to have evidence of neuroendocrine tumor. The resulting aberrant intestinal crypt compartment would have potential to profoundly enhance intestinal tumorigenesis, reflected in the phenotype seen in the studied family. In the intestinal tumors, *WNT2* expression was abundant in the adenomas and carcinomas of the deletion carriers, but not detected in SI-NET tissue. This could be due to silencing of the *CFTR* enhancer function during neuroendocrine tumor development, a hypothesis requiring further validation.

The deletion, removing the first exon of *CFTR*, inactivates the affected gene copy. This renders the deletion carriers also as unaffected cystic fibrosis carriers. One manifestation of cystic fibrosis is increased risk of gastrointestinal cancers (18,19). The standard incidence ratio (SIR) has been reported to be particularly high for gallbladder/extra hepatic bile duct cancers (SIR: 31.9, 95% CI: 1.6–159.0) and small intestinal cancers (SIR: 52.5, 95% CI: 8.8–175.0) in transplanted cystic fibrosis patients (18). Although the risks are high, manifestation of these tumors are rare, and in the largest study reported to date, with 41 188 cystic fibrosis patients, only five small intestinal and four gallbladder/extra hepatic bile duct tumors were reported (19). Detailed histology was described only for one of the small intestinal cancers, and it was reported to be carcinoid tumor (neuroendocrine tumor) of the terminal ileum (18). Interestingly, heterozygous *CFTR* (F508del) mutation carrier status has been recently associated with increased risk for colorectal and gallbladder/biliary tract cancer, but not with small intestinal cancer (20). Furthermore, evidence exists that *CFTR* is a potential tumor suppressor gene for mouse intestinal and human colorectal cancers (21). Taken together, it is not possible to unambiguously exclude that in addition to *WNT2*, *CFTR* or other genetic modifiers, would contribute to the observed phenotype. Whether the vicinity of *WNT2* locus contributes to intestinal cancer predisposition in cystic fibrosis mutation carriers remains to be examined.

While most genes involved in Mendelian disease predisposition may have been identified, structural variation in TAD borders has the potential to lead to previously uncharacterized familial high-penetrance disease phenotypes. The existence of this type of pathogenic variation speaks for utilization of whole genome sequencing in diagnostic efforts tackling apparently Mendelian conditions that are resistant to more limited approaches. The results also emphasize the potential of the emerging WNT inhibitors in management of intestinal neoplasia (22).

## Materials and Methods

The study was approved by the Finnish Institute for Health and Welfare (THL; 151/5.05.00/2017) and the Ethics Committee of the Hospital district of Helsinki and Uusimaa. All participants who donated fresh tissue samples signed an informed consent. The use of archival diagnostic specimens was authorized by the National Supervisory Authority for Welfare and Health (Valvira; 1423/06.01.03.01/2012). The samples and their use in different experiments are summarized in the Supplementary Material, Table S4.

### Genetic analyses

Genotyping was performed using Illumina HumanOmni2.5 v1.0 SNP chips (Illumina), at the Institute for Molecular Medicine Finland (FIMM [Helsinki, Finland]). Illumina Genomestudio v.2.0 (Illumina) was used for genotype calling. Genotypes with a GenCall score < 0.15 were excluded. The CEPH population in the HapMap phase II dataset was used to determine allele frequencies (23) and genotypes with MAF ≥0.1 were selected for the linkage analysis.

Parametric multipoint linkage analysis, with an autosomal dominant inheritance model was performed with Merlin (v.1.1.2) (24). In the analysis, all genotyped SI-NET patients (III-6, III-7, IV-2, IV-8, IV-9, V-2) and the obligatory mutation carrier with colorectal adenocarcinoma (III-4) were marked as ‘affected’. Individuals I-1, I-2, II-1, II-4, II-5, III-1, IV-3, IV-4 and IV-6 were marked as ‘missing phenotype’, and the spouses of II-1, III-4, III-6, III-7 and IV-8 as ‘unaffected’ (Supplementary Material, Fig. S5). Merlin’s error detection algorithm and pedwipe command was utilized to remove unlikely genotypes. Marker distances in cM were determined based on the HapMap phase II genetic map (23), and the parametric LOD scores calculated for equally spaced locations (option —grid 0.25) along the chromosomes. Chromosomal regions with positive LOD score were determined and regions within 1 cM distance were merged.

Whole genome sequencing libraries were prepared using an Illumina TruSeq PCR-free library prep kit, and sequenced (paired-end 150 bp reads) by Illumina HighSeq X Ten at SciLife laboratory (Stockholm, Sweden). The reads were aligned with Burrows-Wheeler Aligner (BWA)–MEM version 0.7.12 (25) against the GRCh37 genome from the 1000 Genomes Project (human_g1k_v37.fasta). The variants were called with HaplotypeCaller following GATK (v3.5) best practices (26,27). The in-house whole genome sequencing pipeline is described in more detail in (28). The mean coverage of the genomes ranged from 30.7–32.3, and the number of mapped reads exceeded 98.6%. Comparative variant analysis, polymorphism filtering, annotation and variant visualization was performed in BasePlayer (29), with Ensembl GRCh37 (release 84) genomic annotation. Variants were required to have ≥10 read coverage and ≥ 25% variant allele frequency, and excluded all the variants residing outside the ‘Strict’ accessibility mask regions from 1000 Genomes phase 3 (30). We also exclude variants with MAF > 0.001 in the gnomAD, V2.0.1; http://gnomad.broadinstitute.org/) and in the Finnish subset of gnomAD (*n* = 12 897).

Structural variants (SVs) were called using DELLY (v0.09) (31,32) as described in (33). SVs that we considered further had to be supported by at least three read pairs (34) and were not allowed to be present in any of the 327 Finnish in-house control whole genomes. We also excluded the SVs that resided outside positive LOD score regions, or were not present in all four whole genome sequenced affected individuals.

### PCR and sanger sequencing and cDNA synthesis

PCRs were performed using AmpliTaq Gold DNA polymerase (Applied Biosystems by Life Technologies). Products were run on an agarose gel and purified using the A’SAP PCR cleanup kit (Arctic Zymes) and Sanger sequenced at FIMM. Sequences were analyzed manually and with Mutation Surveyor (v4.08) (Softgenetics). PCRs from archival samples were performed in at least three replicates. For cDNA synthesis, RNA was reverse transcribed using random primers and Moloney Murine Leukemia Virus (M-MLV) reverse transcriptase (Promega). The primers are listed in Supplementary Material, Table S9 and were designed with Primer3web (v4.0.0) (34,35).

### RNA sequencing

Tissues were homogenized using Ultra Turrax® (IKA) and RNA was extracted with the Qiagen RNeasy mini kit (Qiagen). Sequencing libraries were prepared from total RNA (1 μg) using the KAPA Stranded RNA-seq kit with RiboErase (Roche) and paired-end sequenced (86 bp or 100 bp reads) with HiSeq4000 at Karolinska Institutet (Stockholm, Sweden), or with HiSeq2500 at FIMM. All samples, regardless of their RNA integrity numbers, were used for library preparation and sequencing (Supplementary Material, Table S10). Data from four small intestine and five colon tissue samples, obtained from https://www.ebi.ac.uk/arrayexpress/ (accession code E-MTAB-1733 (25) were used as controls (Supplementary Material, Table S4).

Raw reads were quality and adapter trimmed with cutadapt (v1.16) in Trim Galore (v0.5.0) (36). Low-quality read-ends were removed using a Phred score cut-off of 30. Adapters were trimmed using the first 13 bp of the standard Illumina paired-end adapters. Trimmed reads were HISAT2 (v2.1.0) aligned to gencode.v27lift37.annotations. StringTie (v1.3.4) was used to calculate the coverages at the transcripts (Supplementary Material, Table S10). Statistical testing was performed for small intestine and colon sample sets separately using DESeq2 (v1.14.1). Two designs were used: (i) ~ group + individual (ii) ~ group + genotype. Group was defined as normal or tumor for small intestine samples and as normal, hyperpolyp or adenoma for colon samples. Tumor versus normal test results are from design (i) and Del versus Wt results are from design (ii).

### Single cell gene expression analysis

Fresh tissue samples (Supplementary Material, Table S4) were dissociated with gentleMACS™ Octo Dissociator with heaters (Miltenyi Biotec) using the human Tumor Dissociation Kit (#130–095-929, Miltenyi Biotec), following the manufacturer’s protocol ‘Dissociation of soft tumors’. Cell suspensions were strained with a 70 μm cell strainer and centrifuged at 300rcf for 5 min. Red blood cells were lyzed by incubating in the ACK lysing buffer for 5 min. Cells were washed (centrifugation at 300rcf for 5 min) and diluted in 1× PBS (phosphate-buffered saline) with 0.04% BSA (bovine serum albumin) and filtered through a 40 μm Flowmi™ Tip strainer. Cell count and viability was measured by LUNA-FL™ Dual Fluorescence Cell Counter (Logos Biosystems). The Single Cell 3’RNAseq library preparations were done using the Chromium™ Single Cell 3’ Reagent 2 chemistry and sequenced on Illumina NovaSeq 6000 with read lengths: 26 bp (Read 1), 8 bp (i7 Index), 0 bp (i5 Index) and 91 bp (Read 2) at FIMM.

Data processing and analysis were performed using 10x Genomics Cell Ranger (v2.1.1) pipelines. Cell Ranger ‘cellranger mkfastq’ was used to produce FASTQ (raw data) files and ‘cellranger count’ to align, filter and count unique molecular identifiers (UMIs); mkfastq was run using Illumina bcl2fastq (v2.2.0) and reads aligned against human genome GRCh38. The processed data was plotted in R (v3.3.3) using cellrangerRkit (v1.1.0). For heatmaps, the five most up-regulated genes in each k-means 10 cluster were prioritized based on *P*-value. Only genes with mean normalized UMI counts per cell exceeding 0.5 were considered. Before visualization of *WNT2*, *CFTR, OLFM4* and *EPHB2* signature across all cells, the expression of genes with at least one UMI count was normalized for each barcode and log2 transformed. Cluster ID determination and cell counting was performed in Loupe Cell Browser (10x Genomics, v2.0.0). Cluster IDs were determined based on expression of known intestinal cell markers. In the cell counting, all cells with least one unique molecular identifier count were considered.

### RNA *in situ* hybridization

RNA *in situ* hybridization was performed on fresh 5 μm FFPE tissue sections using the RNAscope Multiplex Fluorescent Reagent Kit v2 (Advanced Cell Diagnostics, Inc.). The Hs-WNT2 (#584071, ACD), Hs-CFTR (#603891-C2, ACD), positive control probe mix (3-plex Positive Control Probe- Hs #320861, ACD) or negative control probe mix (3-plex Negative Control Probe #320871, ACD) were hybridized for 2 h at 40°C, followed by signal amplification and developing according to the manual. Tyramide signal amplification (TSA) Plus Cyanine 3 or Cyanine 5 fluorophores (Perkin Elmer) were used at 1:750 and 1:3500 dilutions, respectively. The sections were counterstained with DAPI and mounted with ProLong Gold Antifade Mountant. Representative areas were scanned using 3DHISTECH Pannoramic 250 FLASH II digital slide scanner at Genome Biology Unit (Helsinki, Finland) and a Plan-Apochromat objective with PCO.edge 4.2 camera with 1 × 40 magnification in 7 focus levels, and CaseViewer (v2.2) was used to create images from the scanned slides. Different tissue types were stained and scanned in separate batches. Identical image settings were used in images created from the same staining and scanning batches. Details of the image settings are described in the figure legends.

### Organoid culture

Biopsies were minced and incubated in 10 mM EDTA-PBS solution for 2.5 h on ice during which buffer was changed every 20–30 min. Epithelial crypts were isolated by vigorous shaking and pipetting the tissue with a 10 mL serological pipette. Epithelium was pelleted and washed once with ‘basal media’: Dulbecco’s Advanced DMEM/F12 (Life) containing 10 mM Hepes, penicillin and streptomycin and 1x Glutamax (Life). The epithelial preparation was resuspended in basal media supplemented with 1 × B27 (Life), 1 × N2 (Life), 50 ng/ml EGF (RnD), 100 ng/ml noggin (Peprotech), 500 ng/ml R-spondin1 (RnD), 1 μM N-Acetyl-L-Cystein (Sigma), 500 nM A-83-01 (Sigma), 10 μM SB202190 (Sigma), 1 mM Nicotinamide (Sigma), 10 μM Leu-Gastrin (Sigma), 3 μM Chir99021 (Sigma), hereafter ‘organoid media’ and mixed with Matrigel® (Corning) in 2:3 ratio. About, 20 μL drops of Cell-Matrigel® mixture were plated on a 48-well plate and let to solidify for 15 min at +37°C. Drops were overlaid with 300 μL of organoid media containing 10 μM Y-27632 (Sigma) during the first two days after isolation. Media was changed every 2–3 days. Approximately once a week, organoids were mechanically broken and subcultured in 1:3 ratio. WNT-dependent organoid growth was analyzed from subcultured organoids overlaid with organoid media containing various concentrations of Chir99021 or vehicle (dimethyl sulfoxide [DMSO]), as indicated in respective figures and figure legends. Media was changed after two days and organoid survival assessed after four days in culture.

### Single cell sorting

Grown organoids were collected to 15 mL falcon tubes, spun down and broken by pipetting repeatedly with 200 μL pipette. Organoid fragments were washed once with cold PBS and digested by incubating in 150 μL of TrypLE Express (Life) for 15 min at +37°C. Single cells were isolated from aggregates by pipetting with a 200 μL pipette. TrypLE Express was inactivated and washed away with 2 mL of basal media. Single cells were resuspended in basal media containing a 1:500 dilution of APC conjugated rat anti-human CD44 (clone: IM7, eBioscience). Cells were incubated for 15 min on ice followed by washing with basal media. Cells were labeled with dead cell dye, SYTOX™ Blue (Thermo Scientific), 1:500 in 2% BSA—PBS prior sorting with FACSAria™ Fusion (BD). Viable SYTOX™ Blue^−^CD44^+^cells were sorted and resuspended in organoid media lacking Chir99021 and containing 10 μM Y-27632. Cell suspension was mixed with Matrigel™ and 5 μL drops containing 1000 cells were plated on a 96-well plate and let to solidify for 15 min at +37°C. 100 μL of organoid media lacking Chir99021 and containing 10 μM Y-27632 and supplemented with Chir99021 or IWP-2 (Sigma) as indicated in the respective figures and figure legends was overlaid. Media was changed every 2 days and organoid formation was quantified on Day 6 (Supplementary Material, Fig. S6).

## Supplementary Material

HMG-2021-CE-00288_Aavikko_Online_Supplementary_material_revised_ddab206

HMG-2021-CE-00288_Aavikko_Supplementary_Table_1_revised_ddab206

HMG-2021-CE-00288_Aavikko_Supplementary_Table_2_revised_ddab206

HMG-2021-CE-00288_Aavikko_Supplementary_Table_3_revised_ddab206

HMG-2021-CE-00288_Aavikko_Supplementary_Table_4_revised_ddab206

HMG-2021-CE-00288_Aavikko_Supplementary_Table_5_revised_ddab206

HMG-2021-CE-00288_Aavikko_Supplementary_Table_6_revised_ddab206

HMG-2021-CE-00288_Aavikko_Supplementary_Table_7_revised_ddab206

HMG-2021-CE-00288_Aavikko_Supplementary_Table_8_revised_ddab206

HMG-2021-CE-00288_Aavikko_Supplementary_Table_9_revised_ddab206

HMG-2021-CE-00288_Aavikko_Supplementary_Table_10_revised_ddab206
