# Supplementary material for: WNT2 activation through proximal germline deletion predisposes to small intestinal neuroendocrine tumors and intestinal adenocarcinomas
Source: Hum Mol Genet. 2021 Jul 19;30(24):2429–40. doi: 10.1093/hmg/ddab206 (PMC8643507; doi:10.1093/hmg/ddab206)
Supplement: HMG-2021-CE-00288_Aavikko_Online_Supplementary_material_revised_ddab206 [file hmg-2021-ce-00288_aavikko_online_supplementary_material_revised_ddab206.doc]

# *WNT2* activation through proximal germline deletion predisposes to small intestinal neuroendocrine tumors and intestinal adenocarcinomas

Mervi Aavikko^1,2,3^, Eevi Kaasinen^1,2^, Noora Andersson^4^, Nalle Pentinmikko^5^, Päivi Sulo^1,2^, Iikki Donner^1,2^, Päivi Pihlajamaa^2,6^, Anna Kuosmanen^1,2^, Simona Bramante^1,2^, Riku Katainen^1,2^, Lauri J. Sipilä^1,2^, Samantha Martin^1,2^, Johanna Arola^7^, Olli Carpén^7,8^, Ilkka Heiskanen^9^, Jukka-Pekka Mecklin^10,11^, Jussi Taipale^2,6^, Ari Ristimäki^2,7^, Kaisa Lehti^12,13^, Erika Gucciardo^13^, Pekka Katajisto^5,12,15,16^, Camilla Schalin-Jäntti^17^, Pia Vahteristo^1,2^, and Lauri A. Aaltonen^1,2*^

**Affiliations**

^1^Department of Medical and Clinical Genetics, Faculty of Medicine, University of Helsinki, FI-00014 Helsinki, Finland.

^2^Applied Tumor Genomics Research Program, Faculty of Medicine, University of Helsinki, FI-00014 Helsinki, Finland.

^3^Institute for Molecular Medicine Finland (FIMM), Helsinki Institute of Life Sciences (HiLIFE), University of Helsinki, FI-00014, Helsinki, Finland.

^4^Department of Pathology, Medicum, University of Helsinki, FI-00014 Helsinki, Finland. ^5^Institute of Biotechnology, Helsinki Institute of Life Sciences (HiLIFE), University of Helsinki, FI-00014 Helsinki, Finland.

^6^Department of Biochemistry, University of Cambridge, Cambridge CB2 1GA, United Kingdom.

^7^Department of Pathology, HUSLAB, HUS Diagnostic Center, Helsinki University Hospital and University of Helsinki, 00290 Helsinki, Finland.

^8^Research Program in Systems Oncology, University of Helsinki, FI-00014 Helsinki, Finland.  ^9^Endocrine Surgery, Abdominal Center, University of Helsinki and Helsinki University Hospital, 00290 Helsinki, Finland.

^10^Department of Surgery, Central Finland Central Hospital, 40620 Jyväskylä, Finland. ^11^Faculty of Sport and Health Sciences, University of Jyväskylä, FI-40014 Jyväskylä, Finland. ^12^Department of Microbiology, Tumor and Cell Biology, Karolinska Institute, 171 77 Stockholm, Sweden.

^13^Individualized Drug Therapy Research Program, Faculty of Medicine, University of Helsinki, 00014 Helsinki, Finland.

^14^Department of Biosciences and Nutrition, Karolinska Institutet, 141 83 Huddinge, Sweden.

^15^Faculty of Biological and Environmental Sciences, University of Helsinki, FI-00014 Helsinki, Finland.

^16^Endocrinology, Abdominal Center, University of Helsinki and Helsinki University Hospital, 00290 Helsinki, Finland.

### *Correspondence

Lauri A. Aaltonen, M.D., Ph.D

Professor of Tumor Genomics

Director, Applied Tumor Genomics Research Program & Center of Excellence in Tumor Genetics

Biomedicum Helsinki, PO Box 63 (Haartmaninkatu 8)

FI-00014 University of Helsinki, Finland

Tel: +358-2941-25595

Fax: +358 2941 25610

e-mail: [lauri.aaltonen@helsinki.fi](mailto:lauri.aaltonen@helsinki.fi)

**
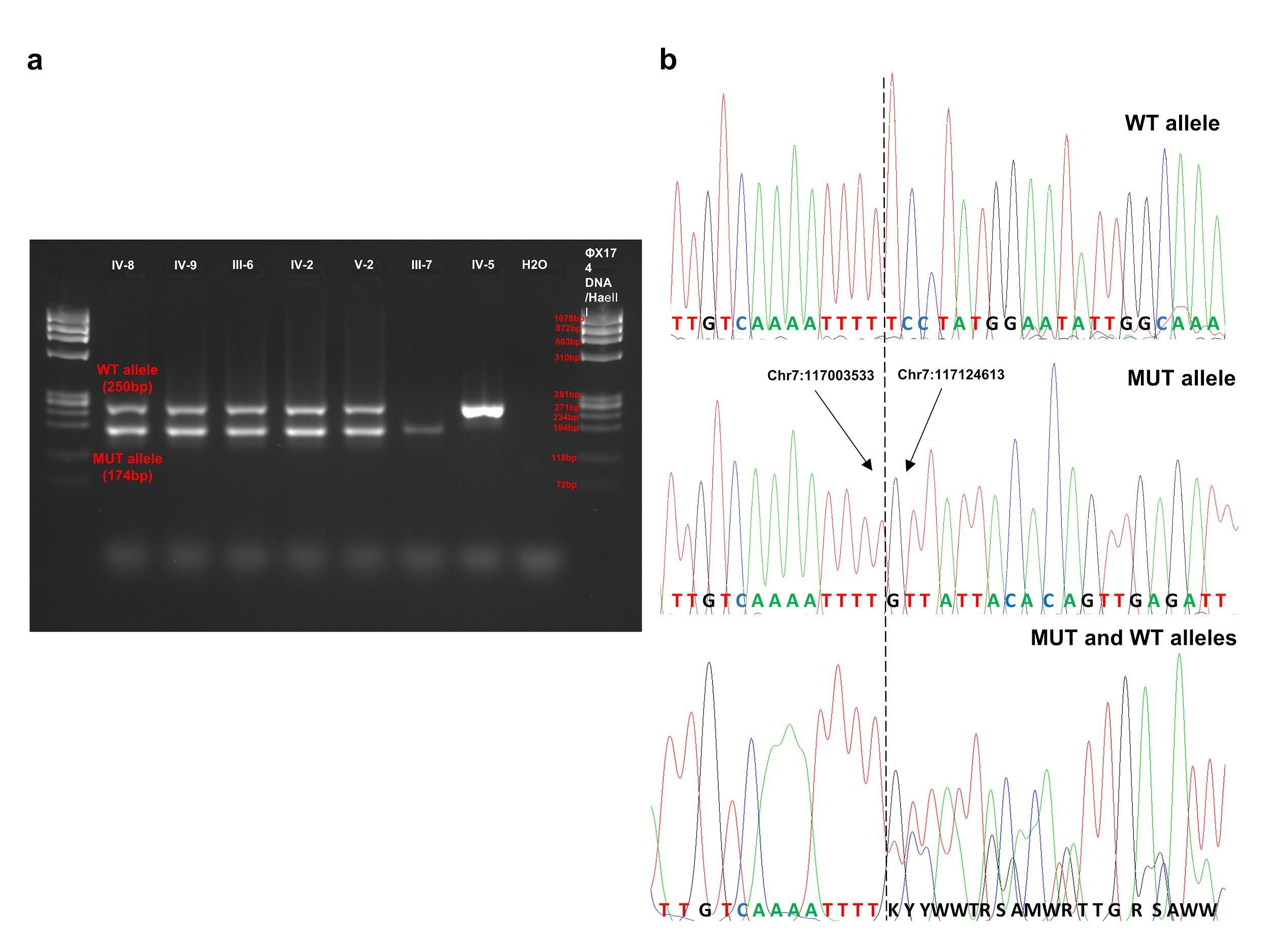
Supplementary Figure 1.** **Determination of the exact sequence of the 7q31.2 deletion.**

**a,** Electrophoresis of the deletion-PCR of six SI-NET patients (IV-8, IV-9, III-6, IV-2, V-2 and III-7) and a healthy family member (IV-5). A mix of three primers (a shared F-primer and allele specific R-primers) were used to amplify the wild type and the deletion allele. The expected amplicon sizes are: mutant (MUT) allele 174bp and wild-type (WT) allele 250bp. Note that from patient III-7 we only had DNA extracted from a formalin fixed paraffin-embedded tissue sample available and for this sample only the mutant fragment is amplified (shorter in length, and thus preferred in PCR). **b,** Representative Sanger sequencing images of a deletion carrier. The uppermost image represents the wild-type (reference) allele sequence. The image in the middle depicts the mutant allele sequence and the image at the bottom describes both the wild type and the mutant allele sequences.


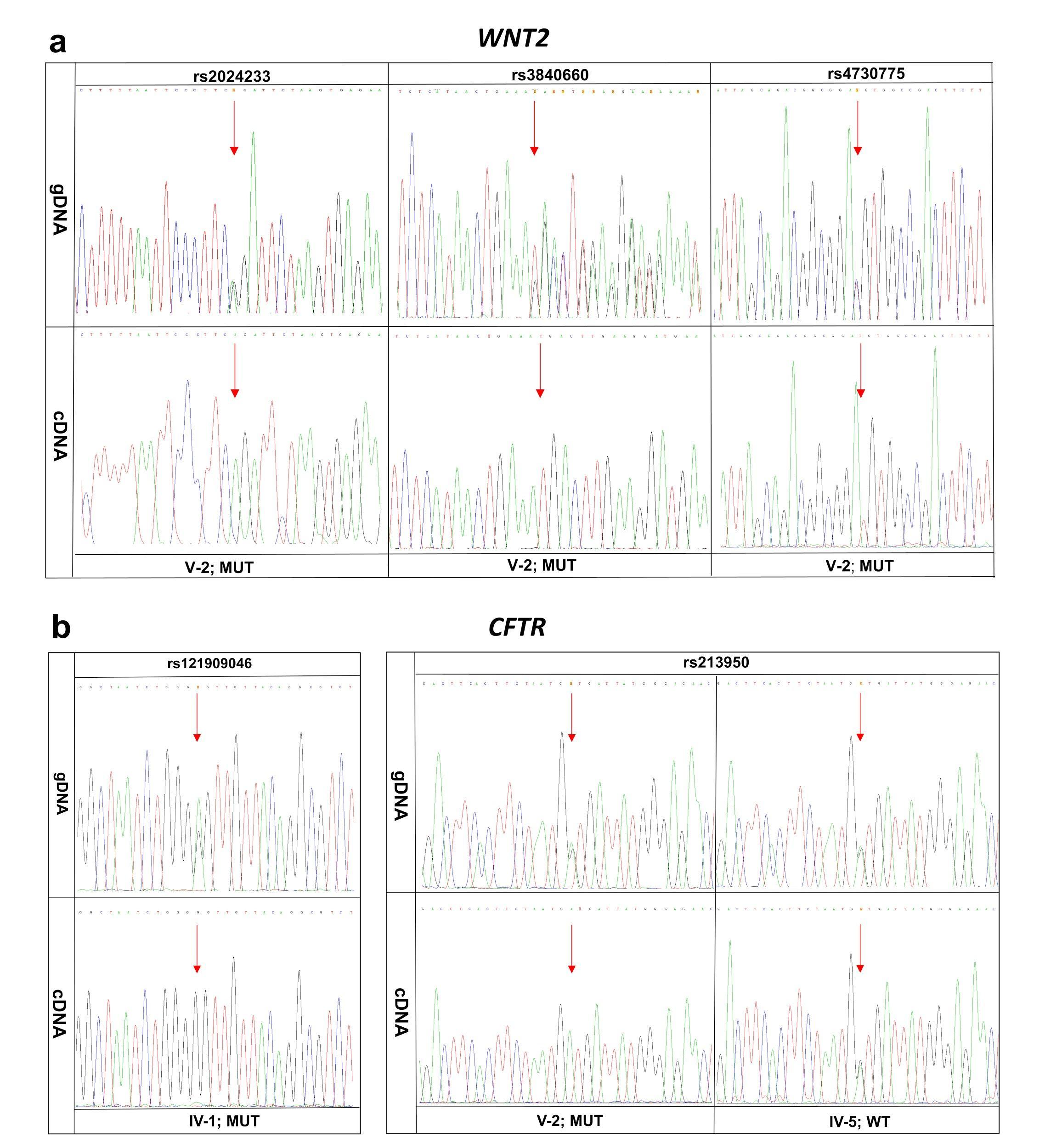


**Supplementary Figure 2. Monoallelic expression of *WNT2* and *CFTR* in the intestine of the deletion carriers*.***

**a,** Representative Sanger sequences from patient V-2 (MUT; mutation carrier) at the site of informative SNPs in *WNT2*. Upper panel: genomic DNA sequences (gDNA) describe heterozygous germline SNPs. Lower panel: cDNA sequences of intestinal samples show expression of only one gene copy of *WNT2.* **b,** Representative Sanger sequences from patients IV-1, V-2 (MUT; mutation carrier) and IV-5 (WT; wild-type allele carrier) at the site of informative SNPs in *CFTR*. Upper panel: genomic DNA sequences (gDNA) describe heterozygous germline SNPs. Lower panel: cDNA sequences of intestinal samples of IV-1 and V-2 (MUT; mutation carriers) show expression of only one copy of *CFTR*, whereas wild-type allele carrier IV-5 shows biallelic expression of *CFTR*.


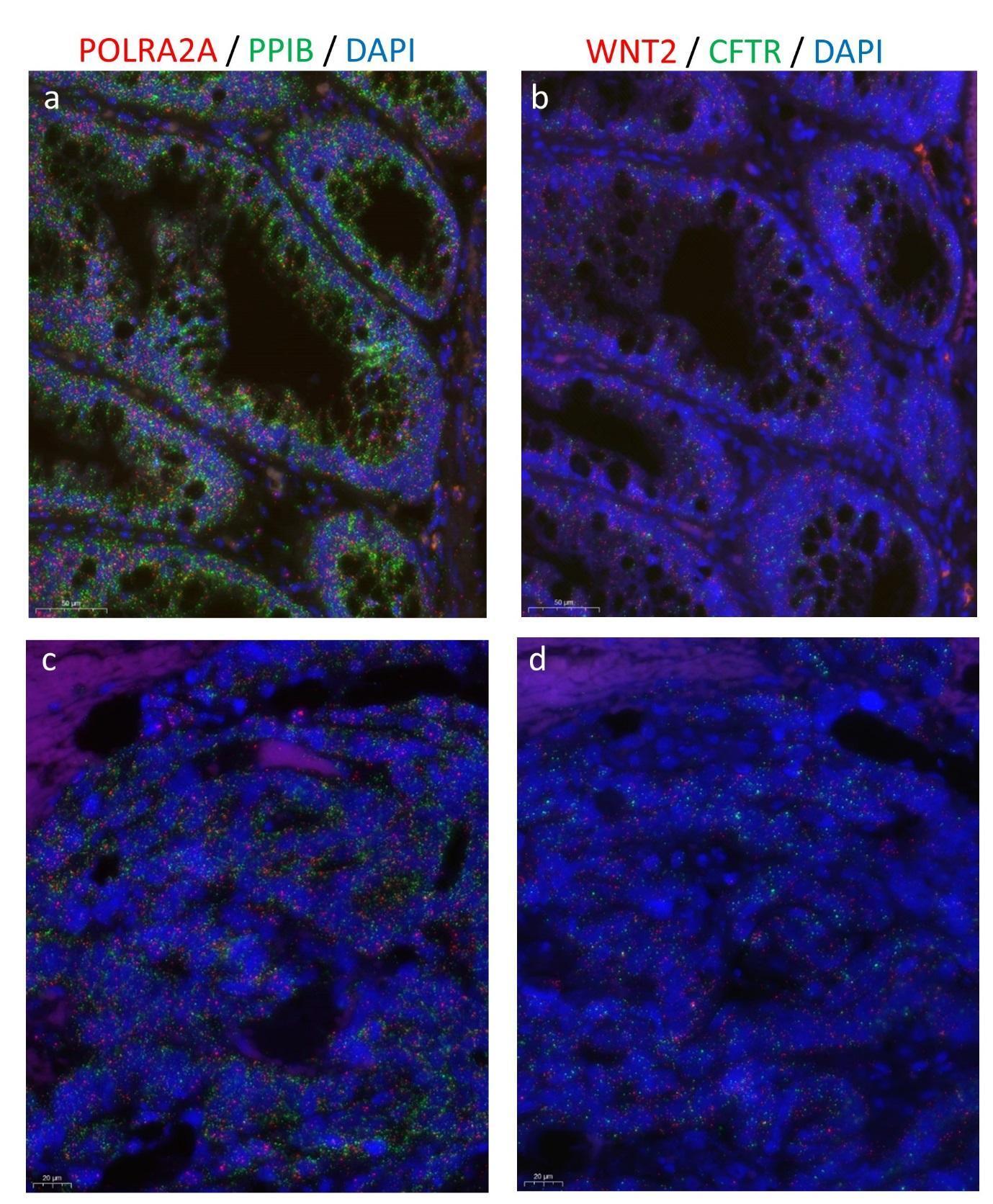
 **Supplementary Figure 3. *WNT2* and *CFTR* are expressed in the colon epithelium and colorectal adenomas of the deletion carriers**.

RNA *in situ* hybridization of a colon epithelium (**a-b**) and an adenoma tissue (**c-d**) of a deletion carrier. The nuclei are stained with DAPI (blue). Left panel: positive control staining with probes against housekeeping genes *POLR2A* (red) and *PPIB* (green). Right panel: *WNT2* (red) and *CFTR* (green) staining. Following intensity settings were used for all images (a-d): DAPI 90, Cy3 150 and Cy5 30.


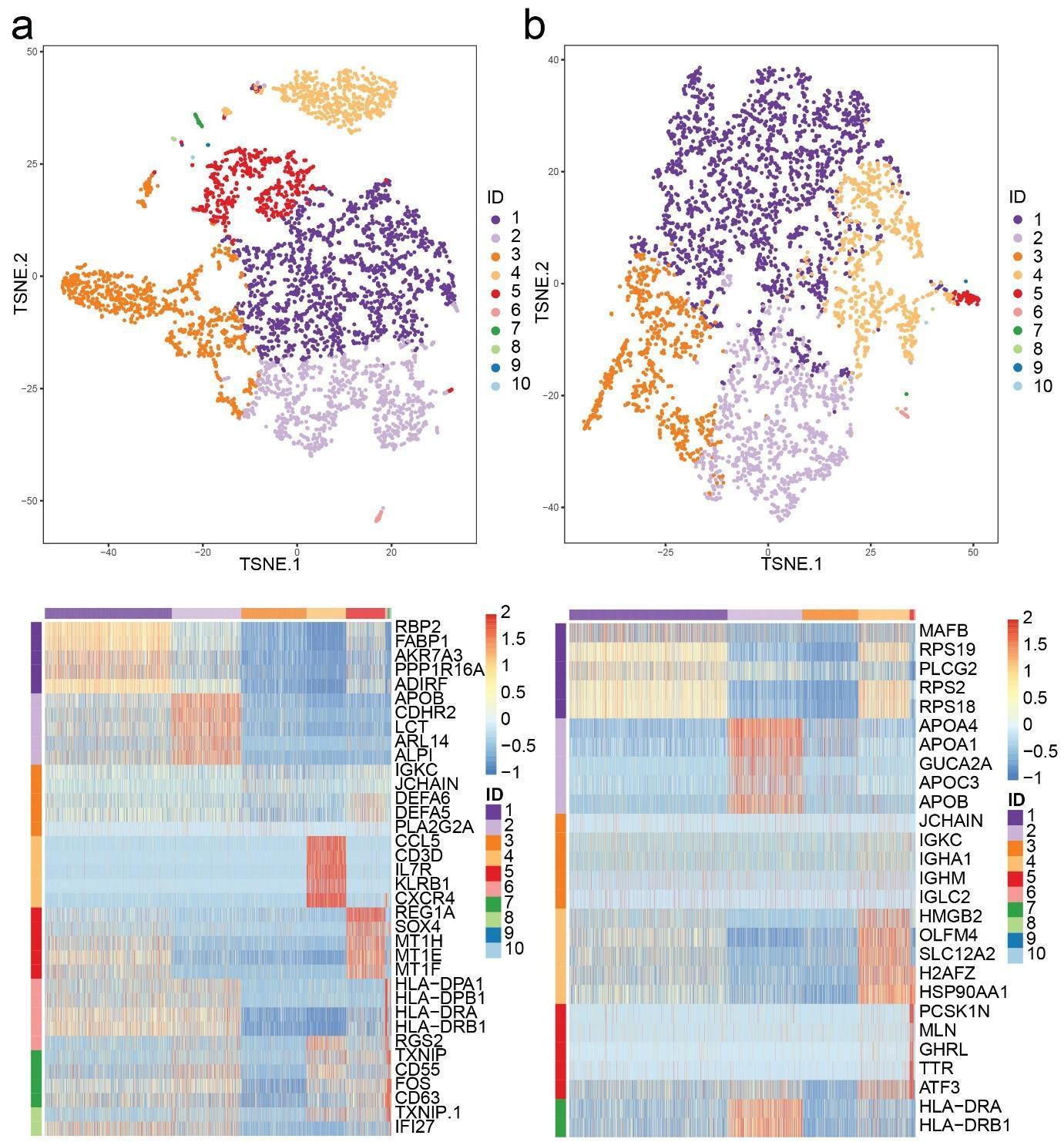


**Supplementary Figure 4. Single cell gene expression analysis of the normal ileum and ileal adenoma specimen of IV-1.**

K-means 10 clustering and heatmap visualization of the five most upregulated genes in each cluster. **a**, Cluster IDs of the normal ileum: 1-2 (epithelial cells), 3 (cells with low gene expression of mainly mitochondrial genes), 4 (immune cells, mainly T and B lymphocytes), 5 (intestinal crypt cells), 6 (complement components expressing cells), 7-8 (miscellaneous), 9 (adipose cells), 10 (muscle cells).

**b**, Cluster IDs of the ileal adenoma: 1-2 (epithelial cells), 3 (immune cells, mainly B lymphocytes), 4 (intestinal crypt cells), 5 (enteroendocrine/secretory cells), 6 (immune cells, likely T lymphocytes), 7-10 (miscellaneous cells). **a-b**, Columns in the heatmap denote cells and rows genes. Color scale represents scaled expression of the genes.


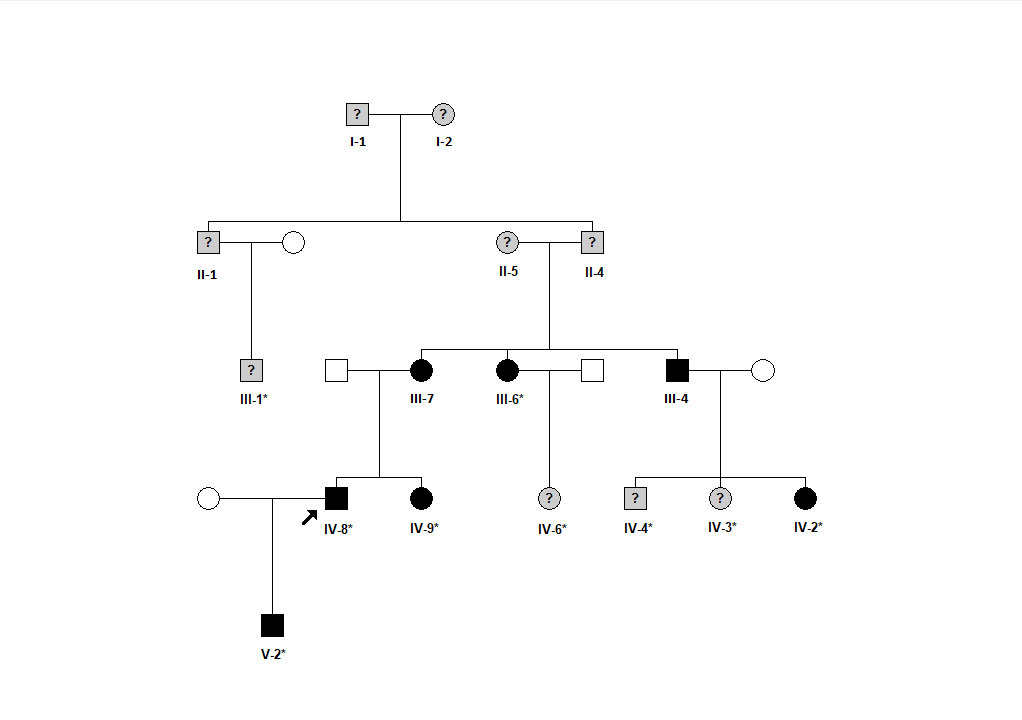


**Supplementary Figure 5. Disease statuses of the family members in the linkage analysis.**

Altogether 21 family members were defined in the linkage analysis and nine of them had SNP chip genotypes available (denoted with asterisks). Squares denote males and circles females. The disease statuses used in the analysis are marked with black (“affected”), grey (“missing phenotype”) and white (“unaffected”). The SI-NET patients (III-6, III-7, IV-2, IV-8, IV-9, V-1) and the obligatory mutation carrier with colorectal adenocarcinoma (III-4) were defined “affected”. Note that the seventh SI-NET patient (IV-1, granddaughter of II-1 and daughter of III-1) was not yet diagnosed at the time when the linkage analysis was carried out and thus, individuals II-1 and II-4 were marked with ”missing phenotype” status. The pedigree has been modified for confidentiality.


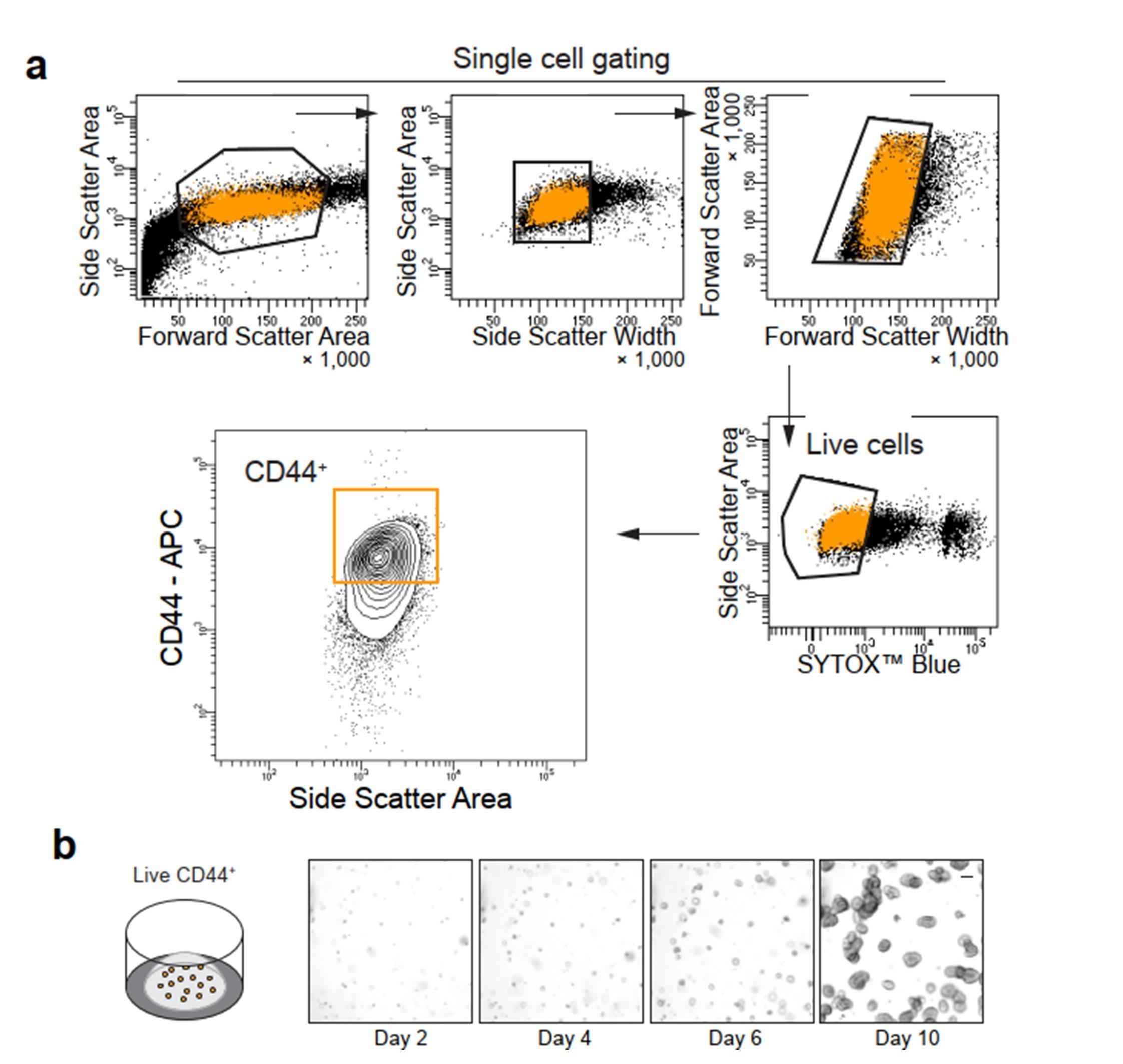


**Supplementary Figure 6. FACS gating strategy. a**, Gating strategy to isolate single live CD44+ cells from dissociated organoids. **b**, Schematic representation of the single cell culture inside 3D Matrigel™. Representative images of organoids forming from single cells derived from V-1 colonic organoids.
